# Investigation of Coagulation Biomarkers to Assess Clinical Deterioration in SARS-CoV-2 Infection

**DOI:** 10.3389/fmed.2021.670694

**Published:** 2021-06-04

**Authors:** Paul Billoir, Kevin Alexandre, Thomas Duflot, Maxime Roger, Sébastien Miranda, Odile Goria, Luc Marie Joly, Mathieu Demeyere, Guillaume Feugray, Valery Brunel, Manuel Etienne, Véronique Le Cam Duchez

**Affiliations:** ^1^Normandie Univ, UNIROUEN, INSERM U1096, CHU Rouen, Vascular Hemostasis Unit, Rouen, France; ^2^F-CRIN INNOVTE, St-Étienne, France; ^3^Normandie Univ, UNIROUEN, EA2656, CHU Rouen, Department of Infectious Diseases, Rouen, France; ^4^Normandie Univ, UNIROUEN, INSERM U1096, CHU Rouen, Department of Pharmacology, Rouen, France; ^5^CHU Rouen, Department of Pneumology, Rouen, France; ^6^Normandie Univ, UNIROUEN, INSERM U1096, CHU Rouen, Department of Internal Medicine, Rouen, France; ^7^CHU Rouen, Department of Gastroenterology and Hepatology, Rouen, France; ^8^Department of Emergency Medicine, Normandie Univ, UNIROUEN, CHU Rouen, Rouen, France; ^9^CHU Rouen, Department of Radiology, Rouen, France; ^10^Department of General Biochemistry, CHU Rouen, Rouen, France

**Keywords:** COVID-19, intensive care, hypercoagulability, fibrinogen, thrombin generation

## Abstract

Since December 2019, a pandemic caused by a new coronavirus has spread to more than 170 countries around the world. Worsening infected patients requiring intensive care unit (ICU) admission associated with 30% of mortality. A part of worsening is induced by hemostasis deregulation. The aim of this study was to investigate the association of coagulation activation in COVID-19 progression. Thirty-five of the 99 patients got clinically worse. The final model of the logistic regression analysis revealed that O_2_ requirement (RR = 7.27 [1.50–19.31]), monocytes below 0.2G/L (RR = 2.88 [1.67–3.19]), fibrinogen levels (RR = 1.45 [1.17–1.82] per g/L increase), prothrombin fragments 1+2 higher than 290 pM (RR = 2.39 [1.20–3.30]), and thrombin peak (RR = 1.28 [1.03–1.59] per 50 nM increase) were associated with an increased risk of clinical worsening. A fibrinogen level threshold of 5.5 g/L, a thrombin peak measurement threshold of 99 pM, and O_2_ requirement associated with clinical outcome in more than 80% of our cohort. In conclusion, we identified fibrinogen and thrombin peak at admission as coagulation biomarkers associated with an increased risk of ICU admission or death. This finding allows initiating steroids and triage for worsening patients. Our results should therefore be considered as exploratory and deserve confirmation.

## Introduction

Since December 2019, a pandemic caused by a new coronavirus has spread to more than 170 countries around the world. It started in China (1) and then spread to Europe and the United States of America. This virus called SARS-CoV-2 (2) is responsible for an infectious disease itself called COVID-19. Most patients are asymptomatic or mildly symptomatic. In symptomatic patients, the clinical manifestations are dominated by respiratory symptoms (2, 3) characterized by serious lung complications that can lead to intensive care unit admission for acute respiratory distress syndrome (4, 5) and to a less extent to cardiovascular injuries (6).

The alteration of the endothelium could originate the deregulation of hemostasis (7). In addition, sepsis promotes platelet overactivation, leading to acute respiratory distress syndrome and acute renal failure (8, 9). Recommendations from the International Society of Thrombosis and Haemostasis (ISTH) and a retrospective study suggest that preventive anticoagulation in patients would be associated with a better prognosis (10, 11).

Prediction models that combine several variables to estimate the risk of people experiencing a poor outcome from the infection could assist medical staff in triaging patients when allocating limited healthcare resources (12). Several scores exist for prediction of mortality in pneumonia such as CURB-65 and A-DROP score (13, 14). Among them, the 4C (Coronavirus Clinical Characterization Consortium) Mortality Score is an easy-to-use and validated prediction tool for in-hospital mortality, accurately categorizing patients as being at low, intermediate, high, or very high risk of death in COVID-19 (AUC = 0.79) (15). However, fewer studies focused on coagulation biomarkers to assess the risk of COVID-19 complications and intensive care unit transfer. Among them, an increase in D-dimer levels has been associated with severe forms of the pathology (16) with other markers of disseminated intravascular coagulation (DIC). Clinical manifestations of these DIC were predominantly thrombotic with high venous thromboembolism rates (5).

Since the beginning of the pandemic, many studies confirmed an increase in D-dimer level (17, 18) and a cutoff value of 2,000 μg/L in patients who were clinically worsening was determined (19). However, D-dimer level is a very sensitive but not a very specific marker of hypercoagulable state. Then, it is possible to assess coagulation globally, by measuring thrombin generation (20). This technique studies the initiation, propagation, and inhibition of coagulation allowing the observation of hypo- or hyper-coagulable risk profiles.

Thus, the aim of this study was to investigate the association of coagulation activation in COVID-19 progression and investigate how coagulation markers could be used to risk stratify patients.

## Methods

### Patients

Between March 16 and May 1, 2020, 100 COVID-19 patients hospitalized in COVID-19 dedicated medical units were prospectively recruited (clinical trials registration number: NCT04367662). An informed consent was obtained from all subjects and citrated plasma from the initial blood test <24 h after the admission was collected, double centrifuged according to French Group of Hemostasis and Thrombosis (GFHT) guidelines, and frozen at −80°C within 4 h after collection. Clinical, radiological, and biological relevant data were also collected. The follow-up of patients were 15 days, with a phone call when a hospital discharged before 15 days.

The study was performed in accordance with the Declaration of Helsinki. The institutional review board (person committee protection of Rouen University Hospital) and a national ethical committee (person committee protection South Mediterranean 1) approved the study, and a national anonymous data collection was declared (Authorization protocol number: 2020-A00914-35).

### Computed Tomography Imaging

As defined by the European Society of Radiology (21), finding COVID-19 pneumonia in computed tomography scan were:

- A scale of disease extension (<10, 10–25%, 25–50%, 50–75%, >75%)
- Condensation type (nodular, linear, or both)
- Radiological abnormalities localization (unilateral, bilateral).

### Assays

During initial blood test, prothrombin time (PT), activated partial thrombin time (aPTT) (DIAGNOSTICA STAGO–Asnières sur Seine, France), and D-dimer (VIDAS DEX2–Biomérieux–Marcy l'étoile, France) assays were performed.

After defrost, several coagulation tests were assayed:

- Fibrinogen (STA-Liquid Fib–DIAGNOSTICA STAGO–Asnières sur Seine, France), Fibrin monomers (STA-Liatest FM–DIAGNOSTICA STAGO–Asnières sur Seine, France), and chromogenic antithrombin assays (StachromATIII–DIAGNOSTICA STAGO–Asnières sur Seine, France) were realized on STA'RMax (DIAGNOSTICA STAGO–Asnières sur Seine, France).
- VWF:GPIb-binding activity (InnovanceVWAc–Siemens Healthcare, Marburg, Germany) was assayed on BCS XP (Siemens Healthcare, Marburg, Germany).
- Prothrombin fragments 1+2 were assayed with Enzygnost F1+2 (Siemens Healthcare, Marburg, Germany) on Diasonrin Etimax.
- Complete blood count was performed on EDTA samples on XN-1000 (Sysmex, Villepinte, France).
- Thrombin generation assay (TGA) was triggered by a low concentration of tissue factor (TF) (1 pM) and a normal concentration of phospholipids (PPP low reagent, Diagnostica Stago, Asnières sur Seine, France). TGA was measured by Calibrated Automated Thrombography and Fluorocan Ascent Fluorometer (Thermoscientific Labsystems, Helsinki, Finland).

### ISTH Disseminated Intravascular Coagulation Score

Disseminated intravascular coagulation score (DIC) was calculated with ISTH criteria recommendation (22). Briefly, the scoring system included platelet count, prothrombin time, fibrinogen, and D-dimer or fibrin monomer.

### Data and Statistical Analysis

The primary objective of the study was to evaluate the association of baseline hemostasis and clinical worsening on admission. Patients were considered to be clinically “worsening” if they were transferred to the intensive care unit or died and clinically “improving” if not. For patient characteristics, data were expressed as median [interquartile range or IQR], *n* (%), or *n*/*N* (%), where *N* is the total number of patients with available data. *P*-values comparing clinical improving to clinical worsening are from χ^2^ test, Fisher's exact test, χ^2^ with *Yates*' *correction* for continuity, Spearman correlation, or Mann–Whitney *U*-test when appropriate. Univariate logistic regression analysis of clinical outcome (improving or worsening) was performed using the following variables as predictors: age, sex, O_2_ requirement, tobacco consumption, radiological scale of disease extension (dichotomized to lower or higher than 25%), body mass index (BMI), hypertension, diabetes, respiratory disease (including COPD and/or asthma and/or other causes of respiratory disease), aPTT ratio (higher than 1.15), blood lymphocyte count (lower than 1 G/L), blood monocyte count (lower than 0.2 G/L), neutrophil-to-monocyte ratio, neutrophil-to-leucocyte ratio, D-dimer (higher than 1,000 μg/L), fibrinogen, TGA parameters (ETP, peak, and velocity), fibrin monomers (higher than 6 μg/ml), VWF: GPIb-binding activity (higher than 250%), and F1+2 (higher than 290 pM). Significant predictors under unadjusted analysis were further analyzed by multiple logistic regression analysis (full model). Then, based on the Akaike Information Criterion (AIC), irrelevant variables were eliminated from the full model by backward variable selection to obtain the final model. Results from the logistic regressions were expressed as relative risk (RR) [95% confidence interval]. Finally, a decision tree based on the predictors retained in the logistic regression final model was built using recursive partitioning method with the following parameters: minimum number of observations that must exist in a node in order for a split to be attempted = 15, minimum number of observations in any terminal node = 5, leave-one-out cross-validation strategy, and complexity parameter that minimizes the cross-validation relative error.

Data and statistical analysis and captions were performed using *R v4.0.0* software (20) and the following software packages: *pROC* (21), *MASS* (22), *caret* (23), *sjstats* (24), *rpart* (25), and *rpart.plot* (26).

## Results

One hundred patients were recruited and followed up to hospital discharge or death. One patient opposed participation after analysis. With World Health Organization classification of COVID-19 severity in admission, 23 patients had pneumonia, 51 patients had severe pneumonia, and 26 patients had acute respiratory distress syndrome. During hospitalization, patients were considered to be clinically worsening (*n* = 35) if they were transferred to the intensive care unit (*n* = 28) or died (*n* = 12) and clinically improving if not. Five patients had anticoagulant treatment before admission for atrial fibrillation. During hospitalization, 46 patients had prophylactic anticoagulation. A second computed tomography was performed in case of respiratory worsening to diagnosis pulmonary embolism. Among them, nine patients developed venous thrombosis: five and three pulmonary embolisms in clinical worsening and improving group, respectively, and one superficial venous thrombosis in clinical improving group. Only one patient who had developed thrombosis did not have thromboprophylaxis. Median follow up was 20.5 days [13–27]. Each patient completed the follow up. No patient developed arterial thrombosis. Demographic and clinical data were reported in Table 1. Age and O_2_ requirement at the time of admission were significantly different between groups. As expected, anticoagulation instauration and hospitalization duration were reported to be significantly different between groups as well as the radiological scale of disease extension.

**Table 1:** Epidemiological, demographic, and clinical characteristics of the 99 hospitalized patients with COVID-19 infection.

**Parameters**	**All (*n* = 99)**	**Improving (*n* = 64)**	**Worsening (*n* = 35)**	***P*-value**
Age, years	65 [51.5–75.0]	63 [48.8–74.3]	71 [59.0–76.0]	**0.044**
Male	53 (54%)	30	23	0.113
Body mass index, kg/m^2^	27.8 [24.0–32.0]	26.9 [23.8–31.0]	28.4 [24.5–32.8]	0.255
**Underlying comorbidity**				
Chronic obstructive pulmonary disease	7 (7.1%)	4 (6.3%)	3 (8.6%)	0.695
Asthma	10 (10.1%)	6 (9.4%)	4 (11.4%)	0.739
Other respiratory disease	9 (9.1%)	7 (10.9%)	2 (5.7%)	0.486
Diabetes	30 (30.3%)	16 (25.0%)	14 (40.0%)	0.186
Hypertension	52 (52.5%)	30 (46.9%)	22 (62.9%)	0.190
Chronic kidney disease (eGFR <30 ml/min)	9 (9.1%)	5 (7.8%)	4 (11.4%)	0.717
Chronic heart failure	4 (4.1%)	2 (3.1%)	2 (5.7%)	0.613
**Previous drug use**				
Immunosuppressant drugs	7 (7.1%)	3 (4.7%)	4 (11.4%)	0.240
**Anticoagulation**				
None	53 (53.5%)	44 (68.8%)	9 (25.7%)	**<0.001**
Standard	25 (25.3%)	13 (20.3%)	12 (34.3%)	
Enhanced	13 (13.1%)	5 (7.8%)	8 (22.9%)	
Curative	8 (8.1%)	2 (3.1%)	6 (17.1%)	
O_2_ requirement on admission	73 (73.7%)	40 (62.5%)	33 (94.3%)	**<0.001**
**Smoking history**				
Never/Former smokers	84 (84.8%)	54 (84.4%)	30 (85.7%)	1.000
Current smokers	15 (15.2%)	10 (15.6%)	5 (14.3%)	
Hospitalization duration	12.0 [6.0–19.0]	9.0 [5.5–14.0]	18.0 [8.5–25.5]	**0.002**
Hospitalization delay since the onset of symptoms	7.0 [4.0–10.0]	7.0 [4.0–10.0]	6.0 [4.0–7.0]	0.195
**Radiological injuries**				
None	3/86 (3.5%)	2/54 (3.7%)	1/32 (3.1%)	**0.009**
Not suggestive	3/86 (3.5%)	3/54 (5.6%)	0/32 (0.0%)	
<10%	11/86 (12.8%)	9/54 (16.7%)	2/32 (6.3%)	
10–25%	39/86 (45.3%)	29/54 (53.7%)	10/32 (31.3%)	
25–50%	15/86 (17.4%)	5/54 (9.3%)	10/32 (31.3%)	
50–75%	13/86 (15.1%)	6/54 (11.1%)	7/32 (21.9%)	
>75%	2/86 (2.3%)	0/54 (0.0%)	2/32 (6.3%)	
**Radiological condensation**				
None	22/82 (26.8%)	17/52 (32.7%)	5/30 (16.7%)	0.427
Nodular	17/82 (20.7%)	10/52 (19.2%)	7/30 (23.3%)	
Linear	37/82 (45.1%)	22/52 (42.3%)	15/30 (50.0%)	
Linear and nodular	6/82 (7.3%)	3/52 (5.8%)	3/30 (10%)	
**Radiological abnormalities localization**				
None	10/81 (12.3%)	8/51 (15.7%)	2/30 (6.6%)	0.554
Unilateral	3/81 (3.7%)	2/51 (3.9%)	1/30 (3.3%)	
Bilateral	68/81 (84%)	41/51 (80.4%)	27/30 (90.0%)	
Thrombosis	8 (8.1%)	3 (4.7%)	5 (14.3%)	0.127
SARS-CoV-2 nucleic acid test pre-admission	81 (81.8%)	50 (78.1%)	31 (88.6%)	0.278

*Data are expressed as median [IQR], n (%), or n/N (%), where N is the total number of patients with available data. Escalated anticoagulation corresponding to 4,000 UI twice a day of enoxaparin, 6,000 UI twice a day of enoxaparin if body weight > 120 kg, or 200 UI/kg of unfractionated heparin. Lymphocytes <1 G/L, monocytes <0.2 G/L, VWF, GPIb-binding activity >250%, Prothrombin fragment 1+2 >290 pM are outside values range. P-values comparing clinical improvement to clinical worsening are from χ^2^ test, Fisher's exact test, or Mann–Whitney U-test. DIC, disseminated intravascular coagulation; ISTH, international society of thrombosis and haemostasis; TGA, thrombin generation assay*.

*Bold values are significant values*.

**Figure 1:**
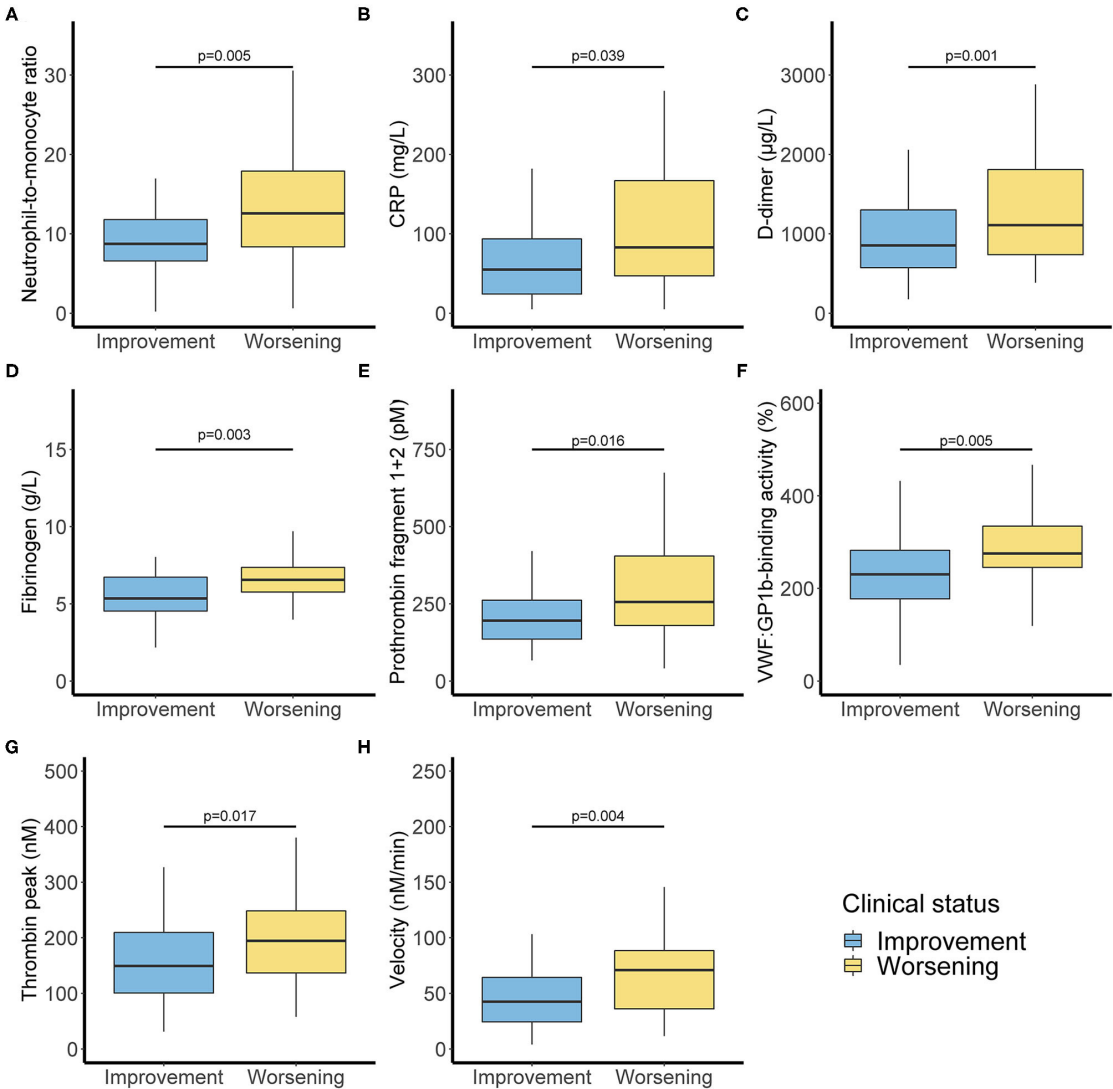
Inflammatory and coagulation biomarkers associated with clinical worsening. Inflammation markers with neutrophil monocyte ratio **(A)** and C-reactive protein **(B)**. Activated coagulation with D-dimer **(C)**, fibrinogen **(D)**, and Prothrombin fragment 1+2 **(E)**. Increased Von Willebrand factor activity **(F)** and thrombin generation with thrombin peak **(G)** and velocity **(H)**. *P*-values comparing clinical improvement to clinical worsening are from Mann–Whitney *U*-test.

In biological markers, we observed a non-significant difference in lymphocyte blood count <1 G/L (50 vs. 67.6%) and a significant difference between clinical worsening and improving for monocyte blood count <0.2 G/L (1.6 vs. 17.6%). Biological characteristics were resumed in Supplementary Table 1. Significant differences are shown in Figure 1. Moreover, neutrophil/lymphocyte ratio was not significantly different (4.4 [2.5–7.5] vs. 5.7 [3.4–11.4]), and we demonstrated that neutrophil/monocyte ratio was increased in worsening group (8.8 [6.7–12.1] vs. 16.7 [9.0–19.8]). Fibrinogen levels and D-dimer were also increased in worsening group. Fibrin monomers and antithrombin levels were not significantly different. ISTH DIC score was calculated at the time of admission either with D-dimer or with Fibrin monomer. We observed a significant difference between worsening and improving patients with ISTH DIC score with D-dimer, and no difference with fibrin monomer scores was significant (2 [2–3] vs. 2 [2–2], and 0 [0–0.25] vs. 0 [0–1], respectively, with D-dimer and Fibrin monomer).

VWF:GPIb-binding activity was also different (*P* < 0.01). Coagulation activation was studied thanks to thrombin generation assay and Prothrombin fragments 1+2 measurement with a significant difference among the two groups (*P* < 0.05).

### Association Between Clinical–Biological Parameters and Clinical Outcome

As described in section 2.5, clinical, radiological, and biological parameters were used as predictors for logistic regression analysis in order to determine predictors of clinical worsening outcome (Table 2). Final model of the logistic regression analysis revealed that O_2_ requirement (RR = 7.27 [1.50–19.31]; *P* = 0.045), monocytes below 0.2 G/L (RR = 2.88 [1.67–3.19]; *P* = 0.015), fibrinogen levels (RR = 1.45 [1.17–1.82] per g/L increase; *P* = 0.005), prothrombin fragments 1+2 higher than 290 pM (RR = 2.39 [1.20–3.30]; *P* = 0.023), and peak of the TGA assay (RR = 1.28 [1.03–1.59] per 50 nM increase; *P* = 0.043) were associated with an increased risk of clinical worsening (Table 2).

**Table 2:** Association factors with clinical worsening (death or intensive care unit admission).

**Patient characteristics**	**Unadjusted^a^**		**Logistic regression full model^b^** **(*****N*** **=** **84)**		**Logistic regression final model^c^** **(*****N*** **=** **84)**	
	**RR (95% CI)**	***P*-value^d^**	**RR (95% CI)**	***P*-value^d^**	**RR (95% CI)**	***P*-value^d^**
Age (per 5-year increase)	**1.10 (1.01–1.19)**	**0.035**	1.00 (0.85–1.17)	0.984		
Sex (Female)	1.67 (0.95–2.48)	0.075				
BMI (per unit increase)	1.03 (0.98–1.08)	0.261				
Chronic respiratory disease	0.82 (0.33–1.48)	0.576				
Diabetes	1.53 (0.87–2.24)	0.123				
HTA	1.53 (0.87–2.29)	0.130				
Oxygenotherapy	**5.88 (2.37–11.0)**	**0.003**	**10.6 (1.87–20.39)**	**0.033**	**7.27 (1.50–19.3)**	**0.045**
Tobacco consumption	0.93 (0.35–1.70)	0.859				
Severe radiological abnormality	**2.73 (1.73–3.55)**	** <0.001**	1.91 (0.64–3.49)	0.210		
Lymphocytes (<1 G/L)	1.64 (0.92–2.49)	0.097				
Monocytes (<0.2 G/L)	**2.79 (1.60–3.22)**	**0.018**	**2.89 (1.46–3.20)**	**0.026**	**2.88 (1.67–3.19)**	**0.015**
NL Ratio (per unit increase)	1.02 (0.98–1.06)	0.300				
NM Ratio (per unit increase)	**1.03 (1.01–1.07)**	**0.023**	0.97 (0.92–1.02)	0.219		
TCA (>1.15)	1.14 (0.61–1.77)	0.652				
D-Dimer (>1,000 μg/L)	**2.12 (1.26–3.03)**	**0.008**	1.58 (0.45–3.15)	0.419		
Fibrin monomers (>6)	1.67 (0.88–2.42)	0.102				
Fibrinogen (per unit increase)	**1.32 (1.13–1.54)**	**0.002**	**1.45 (1.10–1.88)**	**0.020**	**1.45 (1.17–1.81)**	**0.005**
VWF:GPIb-binding activity (>250%)	**2.00 (1.15–2.97)**	**0.021**	0.64 (0.14–1.89)	0.487		
Prothrombin fragment 1+2 (>290 pM)	**2.17 (1.37–2.94)**	**0.004**	**2.58 (1.23–3.46)**	**0.025**	**2.39 (1.20–3.30)**	**0.023**
ETP (per 200 unit increase)	1.12 (0.97–1.27)	0.122				
Peak (per 50 unit increase)	**1.26 (1.07–1.48)**	**0.008**	1.58 (0.59–2.47)	0.308	**1.28 (1.03–1.59)**	**0.043**
Velocity (per 50 unit increase)	**1.75 (1.28–2.20)**	**0.002**	0.64 (0.15–2.63)	0.754		

*Results are expressed as relative risk (RR) (95% confidence interval). N = 84 due to missing data for retained predictors*.

a *Univariate logistic regression analysis*.

b *Multiple logistic regression analysis for variables with P-value below 0.05*.

c *Backward variable selection from the full model*.

d *Wald test*.

*Bold values are significant values*.

**Figure 2:**
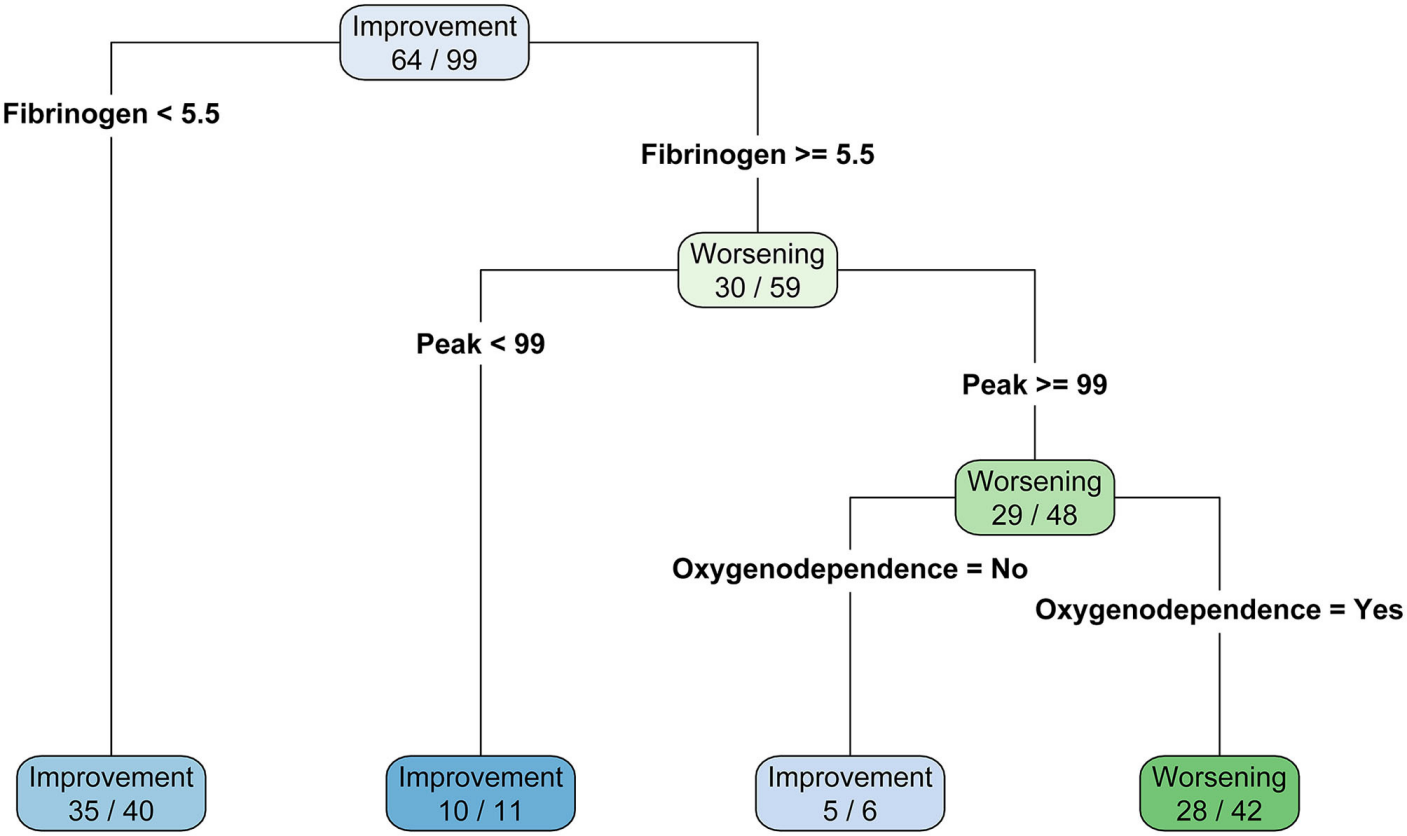
Classification three of clinical status according to final logistic model predictors.

Based on the predictors of the final model of the logistic regression, a classification tree was built in order to establish a hierarchical ranking of predictors to classify patients between clinical worsening and improving. Fibrinogen levels below 5.5 g/L was associated with clinical improving (*N* = 35/40, 87.5%). For patients with higher value than 5.5 g/L, a TGA peak below 99 nM is also predictive of favorable outcome (*N* = 11/12, 90.9%). Then, for patients with fibrinogen higher than 5.5 g/L and TGA peak higher than 99 nM, patients had a better clinical outcome prognosis if they did not depend on O_2_ requirement when compared with patients who need it (*N* = 5/6, 83.3% and *N* = 28/42, 66.7%, respectively). This classification tree provided an accuracy of 79%, a sensitivity of 88%, a specificity of 67%, a positive predicted value of 78%, and a negative predicted value of 80% (Figure 2).

## Discussion

Our study aimed to demonstrate with clinical, radiological, and hemostasis markers the association of clinical worsening in COVID-19 patients. The association between fibrinogen, thrombin peak, and O_2_ requirement had a good correlation with clinical outcome of patients.

Since December 2019, several clinical and biological markers were associated with poor prognosis in COVID-19 patients. Several studies predict the occurrence of critical illness (23–25) and mortality in COVID-19 infection (15, 26–28). Elderly, increased body mass index, hypertension (29), diabetes, and male gender (30) were demographics and associated comorbidity regularly included in the predictive score. As expected, O_2_ requirement in preadmission was a predictive factor to develop worsening SARS-CoV-19. Among biological markers, increases in C-reactive protein and urea are regularly in the prognostic score. C-reactive protein can increase rapidly after the onset of inflammation, cell damage, or tissue injury. The endothelium supports an extensive repertoire of natural anticoagulant. However, during sepsis, activated endothelium increase in TF expression within the vasculature is considered a pivotal step in initiating and sustaining coagulation. The concept of sepsis induced endothelial dysfunction is known as thromboinflammation (31). Few studies evaluated coagulation biomarkers to predict intensive care unit transfer and death in COVID-19. Zhang et al. (19) described an increase of D-dimer associated with poor prognosis. However, the rise of D-dimer during hospitalization is associated with a limited performance to predict death (26). In the study of He et al. (32), the D-dimer cutoff at hospital discharge or death is 2,025 μg/L (AUC: 0.909) and associated with a poor prognosis. In our study, D-dimer >2,000 was not associated with clinical worsening. However, the aim of the study was not the same, with death for He et al. Moreover, the C-reactive protein was more correlated with disease severity compared to D-dimer (33). Our results suggest that fibrinogen had a high discriminate power and a more specific manner than D-dimer does.

Prothrombin fragments 1+2 are less impacted by inflammation than D-dimer (34). We demonstrated increase of prothrombin fragments 1+2. In a recent study evaluating prothrombin fragment 1+2 in COVID-19-associated thrombosis (35), a prothrombin fragment 1+2 >500 pmol/L was associated with venous thromboembolism (odds ratio: 4.26). Conversely, a D-dimer >2,500 ng/mL was not significantly associated with VTE (odds ratio: 5.91).

The interest of global coagulation assay has been previously demonstrated in COVID-19 (36). TGA has already been used to evaluate hypercoagulability (37–40) and acute ischemic stroke development (41). The fact that SARS-CoV-2 virus induces severe endothelial injury associated with intracellular virus and disrupted endothelial cell membranes (42) make TGA an interesting tool to predict clinical outcome of SARS-CoV-2-infected patients. Indeed, microangiopathy and occlusion of alveolar capillaries from lung patients with COVID-19 were found to be secondary to widespread vascular thrombosis (42). The monocytopenia count below 0.2 G/L could be related to COVID-19 severity. This is in accordance with the fact that a decreased monocyte count is associated with poor prognosis in sepsis (43). Recruitment of monocytes is essential for effective control and clearance of viral, bacterial, fungal, and protozoal infections (44). The inflammatory recruitment failure is also a possible explanation to aggravation.

Several studies have described DIC in some COVID-19 patients. In the study of Fogarty et al. (45), DIC was rare and appeared in the late stage disease. In two others studies (16, 46), DIC was significantly more frequent in non-survivors than in survivors. In contrast, in the 24 patients from Panigada's report (47), DIC was not evidenced. With ISTH score, we demonstrated DIC score increase with D-dimer, in worsening patients with more than 75% with a DIC score below 3. With fibrin monomer, more than 75% worsening patients had a DIC score below of 1. Furthermore, the increase of platelet and fibrinogen, associated with normal prothrombin time in our patients, explains the normal DIC score results.

The interest to predict clinical outcome in COVID-19 leads to important increases in the demand for hospital beds and shortage of medical equipment. The urgency of diagnostic and prognostic models can assist quickly the efficient triage of patients in the COVID-19 pandemic (12). Several scores exist for the prediction of mortality in pneumonia, such as CURB-65, A-DROP score, and 4C mortality score (13–15). However, these scores are not suitable to determine intensive care unit transfer. Interestingly, our results demonstrated that the association of fibrinogen level, thrombin peak measurement, and O_2_ requirement was an easy-to-apply model that could predict near than 80% of clinical outcome. Of note, we observed 33/35 patients with O_2_ requirement in the clinical worsening group, among which 26 had fibrinogen level higher than 5.5 g/L and TGA peak higher than 99 nM, suggesting the ability of these last two parameters to predict clinical outcome.

The interest of predictive score to worsening, including intensive care unit transfer during hospitalization, is prompt aggressive treatment, including the initiation of steroids and early escalation to critical care if appropriate (48). A recent study demonstrate that coagulation biomarkers are independent predictors of increased oxygen requirement in COVID-19 patients (49), among them increased fibrinogen and decreased FVIII/VWF:Ag ratio. A study confirmed that D-dimer increase is not associated with intensive care unit transfer (23).

In the study of Panigada et al. (47), von Willebrand factor antigen and ristocetin cofactor activities greatly increased. In the Poissy et al. study (50), factor Willebrand antigen levels seem to be associated with a greater PE risk.

Nevertheless, our study presents several limitations. We have a limited sample size, but the aim of the study was to develop an easy-to-use score to help clinicians. Moreover, our study was prospective and each patient has completed the follow up. Furthermore, we used a robust standardized coagulation test. Finally, our predictive score was computed on our total cohort since it did not appear reasonable to split the data into a training and a test dataset. The validation of our predictive score is required to ensure the reproducibility of the developed mode.

## Conclusion

In conclusion, we identified that high fibrinogen, O_2_ requirement, and thrombin peak at admission were associated with a secondary admission in intensive care unit or death. The score allows the initiation of steroids and triage for worsening patients. Our results should therefore be considered as exploratory and deserve confirmation.

## Data Availability Statement

The raw data supporting the conclusions of this article will be made available by the authors, without undue reservation.

## Ethics Statement

The studies involving human participants were reviewed and approved by Institutional review board (person committee protection of Rouen University Hospital) and a national ethical committee (person committee protection South Mediterranean 1; Authorization protocol number: 2020-A00914-35). The patients/participants provided their written informed consent to participate in this study.

## Author Contributions

PB designed the research, performed analysis, analyzed and interpreted the data, and wrote the manuscript. VL analyzed and interpreted the data and wrote the manuscript. TD critically revised the manuscript and checked the statistical methods and results. KA, MR, SM, OG, LJ, MD, GF, VB, and ME included patients and discussed the obtained results and critically revised the manuscript. All authors read and approved the final version of the manuscript.

## Conflict of Interest

The authors declare that the research was conducted in the absence of any commercial or financial relationships that could be construed as a potential conflict of interest.
