# Supplementary material for: Investigation of Coagulation Biomarkers to Assess Clinical Deterioration in SARS-CoV-2 Infection
Source: Front Med (Lausanne). 2021 Jun 4;8:670694. doi: 10.3389/fmed.2021.670694 (PMC8211892; doi:10.3389/fmed.2021.670694)
Supplement: Supplementary file 1 [file Table_1.DOC]

**Table 1 supplemental.** Biological characteristics of the 99 hospitalized patients with COVID-19 infection

| **Parameters** | **All**  **(n=99)** | **Improving**  **(n=64)** | **Worsening**  **(n=35)** | **P value** |
| --- | --- | --- | --- | --- |
| Blood leukocyte count (G/L) | 6.6 [4.9-9.0] | 5.9 [4.9-8.6] | 7.2 [4.8-9.7] | 0.507 |
| Neutrophil count (G/L) | 5.0 [3.0-7.2] | 4.3 [3.0-6.7] | 5.5 [3.4-8.7] | 0.291 |
| Basophil count (G/L) | 0.01 [0.01-0.03] | 0.01 [0.01-0.03] | 0.01 [0.01-0.03] | 0.541 |
| Eosinophil count (G/L) | 0.00 [0.00-0.05] | 0.01 [0.00-0.07] | 0.00 [0.00-0.00] | **0.001** |
| Lymphocytes count (G/L)  <1 G/L | 0.91 [0.63-1.39]  55/98 (56.1%) | 1.18 [0.68-1.44]  32 (50.0%) | 0.81 [0.53-1.21]  23/34 (67.6%) | 0.080 |
|  |  |  |  | 0.134 |
| Monocyte count (G/L)  <0.2 G/L | 0.41 [0.32-0.62]  7/98 (7.1%) | 0.44 [0.33-0.63]  1 (1.6%) | 0.36 [0.28-0.54]  6/34 (17.6%) | 0.089 |
|  |  |  |  | **0.007** |
| Blood platelets count (G/L) | 230 [155-276] | 228 [155-257] | 192 [161-295] | 0.887 |
| Red blood cell (T/L) | 4.5 [4.1-4.9] | 4.5 [4.1-4.9] | 4.5 [4.1-4.9] | 0.720 |
| Haemoglobin (g/dL) | 13.3 [12.0-14.4] | 13.2 [11.9-14.3] | 13.4 [12.2-14.5] | 0.591 |
| Neutrophil-to-Monocyte ratio | 9.6 [7.4-17.2] | 8.8 [6.7-12.1] | 16.7 [9.0-19.8] | **0.005** |
| Neutrophil-to-Lymphocyte ratio | 4.7 [2.7-7.0] | 4.4 [2.5-7.5] | 5.7 [3.4-11.4] | 0.081 |
| Urea (mM) | 5.7 [3.9-9.8] | 4.85 [-3.63-8.3] | 6.8 [4.9-10.4] | 0.034 |
| C-reactive protein (mg/L) | 61 [22-120] | 52.5 [16.3-93.8] | 74 [43-167] | 0.020 |
| Activated partial thromboplastin time ratio  >1.15 | 1.1 [1.0-1.2]  37/97 | 1.1 [1.0-1.2]  23/63 | 1.1 [1.1-1.3]  14/34 | 0124  0.667 |
| Prothrombin time (sec.) | 13.6 [12.9-14.6] | 13.5 [12.9-14.5] | 13.6 [13.1-14.8] | 0.393 |
| D-dimer (µg/L)  > 500  > 1000  > 1500 | 964 [705-1861]  84  47  34 | 863 [575-1513]  52  24  17 | 1483 [890-3684]  32  23  17 | 0.001  0.245  0.013  0.047 |
| Antithrombin (%) | 103 [98-113] | 104 [98-113] | 101 [96-110] | 0.482 |
| Fibrin monomer  > 6 µg/ml | 2.7 [2.0-4.4]  17 (17.2%) | 2.7 [2.0-3.7]  8 (12.5%) | 2.7 [1.9-6.2]  9 (25.7%) | 0.339  0.165 |
| Fibrinogen (g/L) | 5.9 [4.8-6.9] | 5.4 [4.5-5.7] | 6.6 [5.8-7.4] | **0.003** |
| VWF:GPIb-binding activity (%)  > 250 % | 301 [149-319]  55 (55.6%) | 201 [140-267]  30 (46.9%) | 257 [182-434]  25 (71.4%) | **0.005**  **0.032** |
| Prothrombin fragment1+2 (pM)  > 290 pM | 261 [200-317]  30 (30.3%) | 235 [179-294]  13 (20.3%) | 277 [246-341]  17 (48.6%) | **0.016**  **0.007** |
| ISTH DIC Score  D-Dimer based Score  Fibrin monomer Score | 2 [2-3]  0.00 [0.00-1.00] | 2 [2-2]  0.00 [0.0-0.25] | 2 [2-3]  0.00 [0.00-1.00] | **0.008**  0.059 |
| TGA parameters |  |  |  |  |
| Lagtime (min) | 7.1 [6.3-7.2] | 7.1 [6.3-9.2] | 7.3 [6.7-8.8] | 0.679 |
| ETP (nM.min) | 996 [774-1335] | 990 [718-1237] | 1132 [905-1465] | 0.181 |
| Peak (nM) | 168 [112-224] | 155 [101-210] | 201 [137-248] | **0.017** |
| Velocity (nM/min) | 53 [29-78] | 45 [24-64] | 68 [36-89] | **0.004** |
| Time to Peak (min) | 10.8 [9.6-13.3] | 10.8 [9.8-14.4] | 10.4 [9.6-12.4] | 0.096 |

Data are expressed as median [IQR], n (%), or n/N (%), where N is the total number of patients with available data. Lymphocytes<1 G/L, monocytes<0.2 G/L, VWF:GPIb-binding activity> 250 %, Prothrombin fragment1+2> 290 pM are outside values range. P values comparing clinical improvement to clinical worsening are from χ² test, Fisher’s exact test, or Mann-Whitney U test. DIC: disseminated intravascular coagulation ISTH: International Society Thrombosis and Haemostasis, TGA: Thrombin Generation Assay
