# Supplementary material for: Investigation of Coagulation Biomarkers to Assess Clinical Deterioration in SARS-CoV-2 Infection
Source: Front Med (Lausanne). 2021 Jun 4;8:670694. doi: 10.3389/fmed.2021.670694 (PMC8211892; doi:10.3389/fmed.2021.670694)
Supplement: Supplementary file 3 [file Data_Sheet_2.PDF]

```
library(pROC)
library(rpart)
library(rpart.plot)
library(dplyr)
library(sjstats)
library(MASS)
library(ggpubr)
library(caret)
library(ggplot2)
library("ggsci")


COV<-read.csv2("X:/working directory/COVID.csv")
COV$Age5 <- COV$Age/5
COV$ETP200<-COV$TGAETP/200
COV$Peak50<-COV$TGAPeak/50
COV$Velocity50<-COV$TGAVelocity/50
COV$aPTTthresh<-ifelse(COV$aPTT>1.15,"Yes","No")
COV$DDimthresh<-ifelse(COV$Ddim>500,"Yes","No")
COV$DDimthresh2<-ifelse(COV$Ddim>1000,"Yes","No")
COV$DDimthresh3<-ifelse(COV$Ddim>1500,"Yes","No")
COV$Lymphocytesthresh<-ifelse(COV$Lymphocytes<1,"Yes","No")
COV$Monocytesthresh<-ifelse(COV$Monocytes<0.2,"Yes","No")
COV$FibrinMonomerthresh<-ifelse(COV$FibrinMonomer>6,"Yes","No")
COV$VWFGPIbthresh<-ifelse(COV$VWFGPIb>250,"Yes","No")
COV$ProthrombinFragment1.2thresh<-ifelse(COV$ProthrombinFragment1.2>290,
"Yes","No")
COV$KidneyFailure<-ifelse(COV$MDRD<30,"Yes","No")

COV<- mutate_if(COV, is.character, as.factor)
COV$RadiologicalAbnormalities2<-COV$RadiologicalAbnormalities
levels(COV$RadiologicalAbnormalities2) <- c("up", "up", "down","down",
"down","down","up")
COV$RadiologicalAbnormalities2 = factor(COV$RadiologicalAbnormalities2,
levels(COV$RadiologicalAbnormalities2)[c(2,1)])
###DEMOGRAPHICS###

Improving<-COV[which(COV$Clinical=="Improvement"),]
Worsening<-COV[which(COV$Clinical=="Worsening"),]



wilcox.test(Age~Clinical,COV)
chisq.test(xtabs(~Sex+Clinical,COV,na.action=na.omit),correct=FALSE)
wilcox.test(BMI~Clinical,COV)
fisher.test(xtabs(~COPD+Clinical,COV,na.action=na.omit))
fisher.test(xtabs(~Asthma+Clinical,COV,na.action=na.omit))
fisher.test(xtabs(~Other+Clinical,COV,na.action=na.omit))
chisq.test(xtabs(~Diabetes+Clinical,COV,na.action=na.omit))
chisq.test(xtabs(~HTA+Clinical,COV,na.action=na.omit))
fisher.test(xtabs(~KidneyFailure+Clinical,COV,na.action=na.omit))
fisher.test(xtabs(~CHF+Clinical,COV,na.action=na.omit))
fisher.test(xtabs(~Immunosuppression+Clinical,COV,na.action=na.omit))
fisher.test(xtabs(~Thromboprophylaxis+Clinical,COV,na.action=na.omit))
fisher.test(xtabs(~Oxygenodependence+Clinical,COV,na.action=na.omit))
chisq.test(xtabs(~Tobacco+Clinical,COV,na.action=na.omit))
wilcox.test(HospitalizationLength~Clinical,COV)
wilcox.test(SymptomsOnset~Clinical,COV)
fisher.test(xtabs(~RadiologicalAbnormalities+Clinical,COV,na.action=na.
omit))
fisher.test(xtabs(~Condensation+Clinical,COV,na.action=na.omit))
fisher.test(xtabs(~RadioLocalization+Clinical,COV,na.action=na.omit))
```

```
fisher.test(xtabs(~Thrombosis+Clinical,COV,na.action=na.omit))
fisher.test(xtabs(~PCRCoVID+Clinical,COV,na.action=na.omit))
wilcox.test(Leukocytes~Clinical,COV,alternative="two.sided")
wilcox.test(PNN~Clinical,COV,alternative="two.sided")
wilcox.test(PNB~Clinical,COV,alternative="two.sided")
wilcox.test(PNE~Clinical,COV,alternative="two.sided")
wilcox.test(Lymphocytes~Clinical,COV,alternative="two.sided")
fisher.test(xtabs(~Lymphocytesthresh+Clinical,COV,na.action=na.omit))
wilcox.test(Monocytes~Clinical,COV,alternative="two.sided")
fisher.test(xtabs(~Monocytesthresh+Clinical,COV,na.action=na.omit))
wilcox.test(Platelets~Clinical,COV,alternative="two.sided")
wilcox.test(RBC~Clinical,COV,alternative="two.sided")
wilcox.test(Hb~Clinical,COV,alternative="two.sided")
wilcox.test(NLratio~Clinical,COV,alternative="two.sided")
wilcox.test(NMratio~Clinical,COV,alternative="two.sided")
wilcox.test(aPTT~Clinical,COV,alternative="two.sided")
fisher.test(xtabs(~aPTTthresh+Clinical,COV,na.action=na.omit))
wilcox.test(PT~Clinical,COV,alternative="two.sided")
wilcox.test(Ddimer~Clinical,COV,alternative="two.sided")
fisher.test(xtabs(~DDimthresh+Clinical,COV,na.action=na.omit))
chisq.test(xtabs(~DDimthresh2+Clinical,COV,na.action=na.omit))
chisq.test(xtabs(~DDimthresh3+Clinical,COV,na.action=na.omit))
wilcox.test(Antithrombin~Clinical,COV,alternative="two.sided")
wilcox.test(FibrinMonomer~Clinical,COV,alternative="two.sided")
chisq.test(xtabs(~FibrinMonomerthresh+Clinical,COV,na.action=na.omit))
wilcox.test(Fibrinogen~Clinical,COV,alternative="two.sided")
wilcox.test(ProthrombinFragment1.2~Clinical,COV,alternative="two.sided")
chisq.test(xtabs(~ProthrombinFragment1.2thresh+Clinical,COV,na.action=na
.omit))
wilcox.test(VWFGPIb~Clinical,COV,alternative="two.sided")
chisq.test(xtabs(~VWFGPIbthresh+Clinical,COV,na.action=na.omit))
wilcox.test(DICDdimer~Clinical,COV,alternative="two.sided")
wilcox.test(DICFibrinMonomer~Clinical,COV,alternative="two.sided")
wilcox.test(TGAlagtime~Clinical,COV,alternative="two.sided")
wilcox.test(TGAETP~Clinical,COV,alternative="two.sided")
wilcox.test(TGAPeak~Clinical,COV,alternative="two.sided")
wilcox.test(TGAVelocity~Clinical,COV,alternative="two.sided")
wilcox.test(TGATTP~Clinical,COV,alternative="two.sided")

###BOXPLOT###

p1<-ggplot(data=subset(COV, !is.na(NMratio)), aes(x = Clinical, y =
NMratio)) +
  geom_boxplot(aes(fill = Clinical), alpha = 0.5,outlier.shape=NA)+xlab(
"")+ylab("Neutrophil-to-monocyte ratio")+
  geom_segment(aes(x = 1, y = 31, xend = 2, yend = 31),size=0.8,show.
legend=FALSE)+
  annotate(geom="text",label="p=0.005",x=1.5,y=32.5,size=4)+
  scale_fill_jco()+ # Boxplot fill color
  scale_color_jco()+ # Jitter color palette
  theme_classic()+ylim(0,35)+ theme(plot.title=element_text(hjust=0.5))+
  theme(axis.text=element_text(size=16),axis.title=element_text(size=16
),axis.line = element_line(colour = 'black', size = 1.2),legend.title =
element_text(size = 20),legend.text = element_text(size = 20))
p1$labels$fill <- "Clinical status"
leg <- get_legend(p1)
p1<-p1+theme(legend.position="none")


p2<-ggplot(data=subset(COV, !is.na(CRP)), aes(x = Clinical, y = CRP)) +
  geom_boxplot(aes(fill = Clinical), alpha = 0.5,outlier.shape=NA)+xlab(
"")+ylab("CRP (mg/L)")+
```

```
  geom_segment(aes(x = 1, y = 310, xend = 2, yend = 310),size=0.8,show.
legend=FALSE)+
  annotate(geom="text",label="p=0.039",x=1.5,y=325,size=4)+
  scale_fill_jco()+ # Boxplot fill color
  scale_color_jco()+ # Jitter color palette
  theme_classic()+ylim(0,350)+ theme(plot.title=element_text(hjust=0.5))+
  theme(axis.text=element_text(size=16),axis.title=element_text(size=16
),axis.line = element_line(colour = 'black', size = 1.2),legend.title =
element_text(size = 20),legend.text = element_text(size = 20))
p2$labels$fill <- "Clinical status"
leg <- get_legend(p2)
p2<-p2+theme(legend.position="none")


p3<-ggplot(data=subset(COV, !is.na(Ddimer)), aes(x = Clinical, y =
Ddimer)) +
  geom_boxplot(aes(fill = Clinical), alpha = 0.5,outlier.shape=NA)+xlab(
"")+ylab("D-dimer (µg/L)")+
  geom_segment(aes(x = 1, y = 3100, xend = 2, yend = 3100),size=0.8,show
.legend=FALSE)+
  annotate(geom="text",label="p=0.001",x=1.5,y=3250,size=4)+
  scale_fill_jco()+ # Boxplot fill color
  scale_color_jco()+ # Jitter color palette
  theme_classic()+ylim(0,3500)+ theme(plot.title=element_text(hjust=0.5
))+
  theme(axis.text=element_text(size=16),axis.title=element_text(size=16
),axis.line = element_line(colour = 'black', size = 1.2),legend.title =
element_text(size = 20),legend.text = element_text(size = 20))
p3$labels$fill <- "Clinical status"
leg <- get_legend(p3)
p3<-p3+theme(legend.position="none")


p4<-ggplot(data=subset(COV, !is.na(Fibrinogen)), aes(x = Clinical, y =
Fibrinogen)) +
  geom_boxplot(aes(fill = Clinical), alpha = 0.5,outlier.shape=NA)+xlab(
"")+ylab("Fibrinogen (g/L)")+
  geom_segment(aes(x = 1, y = 15, xend = 2, yend = 15),size=0.8,show.
legend=FALSE)+
  annotate(geom="text",label="p=0.003",x=1.5,y=16,size=4)+
  scale_fill_jco()+ # Boxplot fill color
  scale_color_jco()+ # Jitter color palette
  theme_classic()+ylim(0,18)+ theme(plot.title=element_text(hjust=0.5))+
  theme(axis.text=element_text(size=16),axis.title=element_text(size=16
),axis.line = element_line(colour = 'black', size = 1.2),legend.title =
element_text(size = 20),legend.text = element_text(size = 20))
p4$labels$fill <- "Clinical status"
leg <- get_legend(p4)
p4<-p4+theme(legend.position="none")


p5<-ggplot(data=subset(COV, !is.na(ProthrombinFragment1.2)), aes(x =
Clinical, y = ProthrombinFragment1.2)) +
  geom_boxplot(aes(fill = Clinical), alpha = 0.5,outlier.shape=NA)+xlab(
"")+ylab("Prothrombin fragment 1+2 (pM)")+
  geom_segment(aes(x = 1, y = 750, xend = 2, yend = 750),size=0.8,show.
legend=FALSE)+
  annotate(geom="text",label="p=0.016",x=1.5,y=780,size=4)+
  scale_fill_jco()+ # Boxplot fill color
  scale_color_jco()+ # Jitter color palette
  theme_classic()+ylim(0,900)+ theme(plot.title=element_text(hjust=0.5))+
  theme(axis.text=element_text(size=16),axis.title=element_text(size=16
```

```
),axis.line = element_line(colour = 'black', size = 1.2),legend.title =
element_text(size = 20),legend.text = element_text(size = 20))
p5$labels$fill <- "Clinical status"
leg <- get_legend(p5)
p5<-p5+theme(legend.position="none")


p6<-ggplot(data=subset(COV, !is.na(VWFGPIb)), aes(x = Clinical, y =
VWFGPIb)) +
  geom_boxplot(aes(fill = Clinical), alpha = 0.5,outlier.shape=NA)+xlab(
"")+ylab("VWF:GP1b-binding activity (%)")+
  geom_segment(aes(x = 1, y = 500, xend = 2, yend = 500),size=0.8,show.
legend=FALSE)+
  annotate(geom="text",label="p=0.005",x=1.5,y=520,size=4)+
  scale_fill_jco()+ # Boxplot fill color
  scale_color_jco()+ # Jitter color palette
  theme_classic()+ylim(0,600)+ theme(plot.title=element_text(hjust=0.5))+
  theme(axis.text=element_text(size=16),axis.title=element_text(size=16
),axis.line = element_line(colour = 'black', size = 1.2),legend.title =
element_text(size = 20),legend.text = element_text(size = 20))
p6$labels$fill <- "Clinical status"
leg <- get_legend(p6)
p6<-p6+theme(legend.position="none")


p7<-ggplot(data=subset(COV, !is.na(TGAPeak)), aes(x = Clinical, y =
TGAPeak)) +
  geom_boxplot(aes(fill = Clinical), alpha = 0.5,outlier.shape=NA)+xlab(
"")+ylab("Thrombin peak (nM)")+
  geom_segment(aes(x = 1, y = 400, xend = 2, yend = 400),size=0.8,show.
legend=FALSE)+
  annotate(geom="text",label="p=0.017",x=1.5,y=420,size=4)+
  scale_fill_jco()+ # Boxplot fill color
  scale_color_jco()+ # Jitter color palette
  theme_classic()+ylim(0,500)+ theme(plot.title=element_text(hjust=0.5))+
  theme(axis.text=element_text(size=16),axis.title=element_text(size=16
),axis.line = element_line(colour = 'black', size = 1.2),legend.title =
element_text(size = 20),legend.text = element_text(size = 20))
p7$labels$fill <- "Clinical status"
leg <- get_legend(p7)
p7<-p7+theme(legend.position="none")


p8<-ggplot(data=subset(COV, !is.na(TGAVelocity)), aes(x = Clinical, y =
TGAVelocity)) +
  geom_boxplot(aes(fill = Clinical), alpha = 0.5,outlier.shape=NA)+xlab(
"")+ylab("Velocity (nM/min)")+
  geom_segment(aes(x = 1, y = 200, xend = 2, yend = 200),size=0.8,show.
legend=FALSE)+
  annotate(geom="text",label="p=0.004",x=1.5,y=210,size=4)+
  scale_fill_jco()+ # Boxplot fill color
  scale_color_jco()+ # Jitter color palette
  theme_classic()+ylim(0,250)+ theme(plot.title=element_text(hjust=0.5))+
  theme(axis.text=element_text(size=16),axis.title=element_text(size=16
),axis.line = element_line(colour = 'black', size = 1.2),legend.title =
element_text(size = 20),legend.text = element_text(size = 20))
p8$labels$fill <- "Clinical status"
leg <- get_legend(p8)
p8<-p8+theme(legend.position="none")

figbp<-ggarrange(p1, p2, p3, p4,p5, p6, p7, p8,leg,
          labels = c("A","B","C","D","E","F","G","H",""))
```

```
setwd("X:/working directoy/")
png(file = "Fig X.png", width = 12, height = 12, units = 'in',res=600)
figbp
dev.off()




###LOGISTIC REGRESSION###
###UNIVARIATE###
lapply(X = intersect(search(), objects()),
FUN = function(X){detach(name = X, character.only = TRUE)})
covariates<-unlist(strsplit(names(COV), split=" "))
predictors<-covariates[c(54,3,6,7,68,12:15,58,60,43,36,37,55,56,57,62,63
,64,65,66)]

univ_formulas <- sapply(predictors,
                        function(x) as.formula(paste("Clinical~",x)))
univ_models <- lapply(univ_formulas, function(x){glm(x, data = COV,
family=binomial)})
univ_results <- lapply(univ_models,
                       function(x){
                          y <- summary(x)
                          z<-odds_to_rr(x)
                          p.value<-signif(coef(y)[2,4], digits=3)
                          RR <-signif(z[2,3], digits=4)
                          RR5 <-signif(z[2,4], digits=4)
                          RR95 <-signif(z[2,5], digits=4)
                          res2<-c(RR,RR5,RR95,p.value)
                          names(res2)<-c("RR","RR5%","RR95%","p-value")
              return(res2)})
res2 <- t(as.data.frame(univ_results, check.names = FALSE))
res2

###FULLMODEL###
FullModelDF<-COV[,c(23,54,6,68,60,43,37,56,57,63,65,66)]
FullModelDFnoNA<-FullModelDF[complete.cases(FullModelDF),]
FullModel<-glm(Clinical~.,binomial,FullModelDFnoNA)
                          y <- summary(FullModel)
                          z<-odds_to_rr(FullModel)
                          p.value<-signif(coef(y)[2:12,4], digits=3)
                          RR <-signif(z[2:12,3], digits=4)
                          RR5 <-signif(z[2:12,4], digits=4)
                          RR95 <-signif(z[2:12,5], digits=4)
                          FullModelRes<-cbind(RR,RR5,RR95,p.value)
FullModelRes

###FINALMODEL###
FinalModel<-stepAIC(FullModel)
                          y <- summary(FinalModel)
                          z<-odds_to_rr(FinalModel)
                          p.value<-signif(coef(y)[2:6,4], digits=3)
                          RR <-signif(z[2:6,3], digits=4)
                          RR5 <-signif(z[2:6,4], digits=4)
                          RR95 <-signif(z[2:6,5], digits=4)
                          FinalModelRes<-cbind(RR,RR5,RR95,p.value)
FinalModelRes


###CLASSIFICATION TREE###
```

```
TreeDF<-COV[,c(23,6,43,50,35,45)]
names(TreeDF)[4]<-"Peak"
names(TreeDF)[6]<-"PT Fragment"
set.seed(123)
CTinitial<-rpart(Clinical~.,data=TreeDF,control=rpart.control(minsplit=
30,minbucket=6,cp=0,xval=9))
plotcp(CTinitial)
CTinitial$cptable[which.min(CTinitial$cptable[,4]),1]
CTfinal <- prune(CTinitial,cp=CTinitial$cptable[which.min(
CTinitial$cptable[,4]),1])
rpart.plot(CTfinal, type=4,extra=2,clip.right.labs = FALSE,cex=1, main=
"Classification tree of clinical status
according to final logistic model predictors", sub="Accuracy=79%,
Sensitivity=88%, Specificity=67%, PPV=78%, NPV=80%")

confusionMatrix(table(COV$Clinical, predict(CTfinal , TreeDF, type=
"class")))

setwd("X:/working directoy/")
png(file = "Fig XX.png", width = 9, height = 9, units = 'in',res=900)
rpart.plot(CTfinal, type=4,extra=2,clip.right.labs = FALSE,cex=1, main=
"Classification tree of clinical status
according to final logistic model predictors", sub="Accuracy=79%,
Sensitivity=88%, Specificity=67%, PPV=78%, NPV=80%")
dev.off()
```
